# For what factors should we normalize urinary extracellular mRNA biomarkers?

**DOI:** 10.1016/j.bdq.2019.100090

**Published:** 2019-04-23

**Authors:** Pradeep Moon Gunasekaran, James Matthew Luther, James Brian Byrd

**Affiliations:** a Department of Internal Medicine, University of Michigan Medical School, Ann Arbor, MI, USA; b Department of Medicine, Vanderbilt University School of Medicine, Nashville, TN, USA

**Keywords:** Extracellular RNA, Extracellular vesicles, Exosomes, mRNA, Urine, Urinary, Biomarkers, Cardiometabolic, Cancer, ExRNA, Creatinine, Specific gravity

## Abstract

mRNA is a critical biomolecule involved in the manifestation of the genetic code into functional protein molecules. Its critical role in the central dogma has made it a key target in many studies to determine biomarkers and drug targets for numerous diseases. Currently, there is a growing body of evidence to suggest that RNA molecules around the size of full-length mRNA transcripts can be assayed in the supernatant of human urine and urinary extracellular mRNA could provide information about transcription in cells of urogenital tissues. However, the optimal means of normalizing these signals is unclear. In this paper, we describe relevant first principles as well as research findings from our lab and other labs toward normalization of urinary extracellular mRNA.

## 1 Introduction

Several species of RNA including messenger RNA (mRNA) are measurable in the supernatant of human urine [1]. The existence of measurable urinary RNA in a cell-depleted aliquot of urine is potentially surprising. Urine is rich in ribonucleases [2], and mRNA experimentally introduced into urine is rapidly degraded [3]. The RNA measurable in urine is likely shielded from degradation by a mixture of carriers, including extracellular vesicles such as exosomes and microvesicles, ribonucleoproteins, and lipoproteins [4]. The contribution of each of these carriers to the total extracellular RNA content of urine is not yet well understood. Not only has long RNA in the size range of full-length mRNA been extracted from urinary extracellular vesicles [5], mRNA present in an ultracentrifugation-derived pellet of extracellular vesicles from human urine has also undergone massively parallel sequencing, aligning to approximately 13,500 genes [1]. Using this approach, Miranda and colleagues were also able to show the presence of a variety of other RNA species such as noncoding RNA (lncRNA), ribosomal RNA (rRNA), and mitochondrial RNA (mito-RNA). Of the RNA species present in urine, mRNA is of special interest to us due to existing detailed annotations and a relatively advanced state of knowledge regarding its physiological significance. For the purposes of this article, we will describe mRNA shielded by carriers such as extracellular vesicles in the supernatant of urine as urinary extracellular mRNA. We will be focusing our attention on the mRNA isolated from extracellular vesicles, which has been shown to be robustly detectable [1,5,6]. Urinary extracellular vesicle-associated RNA has been sequenced (with the caveat that RNA shuttled by non-vesicular carriers is technically difficult to exclude in such studies). This RNA has been explored as a potential biomarker with promising results [5,7,8], and a company is offering a clinical assay they describe [9] as a “urine exosome gene expression assay.” Yet if there is a generalizable best approach to normalizing an extracellular mRNA signal in the urine, it has not yet been identified.

Normalization of a biomarker can be considered in terms of a numerator and a denominator. The numerator is the measured quantity of the biomarker of interest (which could be a composite signal from more than one molecule). Several different principles could be invoked to identify the optimal denominator, the focus of this article. This article focuses on explaining the first principles we find relevant to the question of normalizing urinary extracellular mRNA biomarkers.

## 2 For what factor(s) should we be normalizing?

**Fig. 1:**
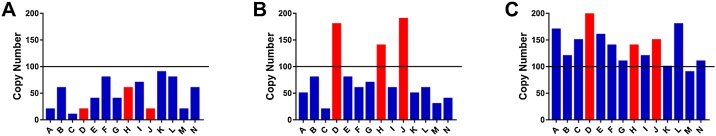
Lack of normalization can lead to the inappropriate conclusions regarding diagnostic assays of urinary extracellular mRNA. A) Artificially generated data to show an idealized illustration of mRNA copy number for target genes (Red) and genes not affected by the disease (Blue) in a healthy individual's urine. B) Artificially generated data illustrating an individual with a pathology which leads to the upregulation of the mRNA of target genes in urine beyond a diagnostic threshold (black line). C) Artificially generated data intended to illustrate an individual with a condition which increases the mRNA levels in urine globally. In this situation, measuring target genes without appropriate normalization could lead to the individual being misclassified as have disease (For interpretation of the references to colour in this figure legend, the reader is referred to the web version of this article).

There are at least two hypothetical sources of confounding variation that might be important denominators when normalizing assays of urinary extracellular mRNA transcripts. The first is changes in the composition of the biofluid matrix broadly affecting analytes’ concentrations (i.e., intra-individual changes or inter-individual differences in urine osmolality due to having recently consumed water). The second is the effect of physiology or pathophysiology on the overall number of extracellular vesicles and other RNA carriers shuttled into the urine in a given period of time. For example, there is a large body of evidence which shows that in various cancers [10], the rate of extracellular vesicle production is upregulated. If this increased secretion of vesicles leads to more vesicles entering the urine, an increased rate of shuttling of RNA into the urine would also be expected. Depending on how the altered secretion rate of vesicles affects the ratio of biomarker molecules to molecules used in normalization, a change in vesicle secretion rate could increase or decrease the signal-to-noise ratio (Fig. 1). The extent to which each of these factors must be taken into account during normalization will depend on the magnitude of target genes’ signal in the disease relative to the magnitude of the noise created by these potentially confounding factors.

### 2.1 Normalizing for changes in urine osmolality (sometimes described in terms of variable urine flow rate)

The normalization of soluble proteins in the urine has often used urinary creatinine in the denominator. The first principles [11] underlying this now evidence-based strategy for normalizing urinary albumin excretion are worth considering for their potential relevance to normalizing urinary mRNA.

#### 2.1.1 Using time to normalize for changes in urine osmolality

Collecting urine specimens over a period of time (e.g., 24 h) mitigates against differences in analyte concentration driven solely by a recent episode of fluid gain (e.g., consuming water) or loss (e.g., sweating). The appropriateness of the amount of albumin excreted into the urine in a 24-hour period can be judged relative to the amount excreted in that same time period by healthy individuals. Although the choice of any discrete time period is arbitrary, the choice of a 24-h period may be expected to provide an integrated measure during most typical activities of daily life which occur regularly over this period. However, collecting every urine specimen for 24 h presents practical difficulties for patients, and the possibility of a pseudo-normal value due to partial collection is a concern. The completeness of the collection cannot be judged based solely on the volume of urine since that varies according to body size, water intake and insensible losses (e.g., perspiration), and renal function, among other factors. Fortuitously, creatinine has properties that make it a useful marker of the completeness of a 24-h urine collection. Creatine is secreted by muscle cells in approximately the same amount within a 24-h period, day after day. It is non-enzymatically converted to the breakdown product creatinine, which is small and thus freely filtered by the renal glomerulus. Creatinine is not absorbed by the renal tubule, and only approximately 10% of the creatinine excreted in the urine comes from tubular secretion. Because common drugs can affect tubular creatinine secretion (e.g., cimetidine, ranitidine, famotidine, and trimethoprim), investigators using creatinine-based normalization of urinary biomarkers should consider inquiring about and standardizing use of these medications during collection. Formulas to predict the amount of creatinine that will be secreted in a 24-h period are available giving investigators a way to judge the *completeness* of the 24-h urine collection [12]. Additionally, the excretion of creatinine has been considered to be a sufficiently robust parameter that it is already widely used in the clinic to normalize the excretion of another urinary analyte, protein. Currently there are multiple ways to determine the concentration of creatinine in a urine sample, such as the classical Jaffe reaction [13] (creatinine and picric acid react under alkaline conditions to form a chromogenic complex which can be quantitatively measured using a spectrophotometer). There is also a more efficient and accurate enzymatic test [14,15]. Using a series of coupled enzymatic reactions involving creatininase, creatinase, and sarcosine oxidase, hydrogen peroxide can be generated in proportion to the amount of creatinine present in the sample. This hydrogen peroxide turn reacts with peroxidase to form a colored dye which can be measured at 550 nm. In summary, the total amount of creatinine excreted in 24 h is a reasonable way to judge the completeness of a 24-h urine collection since strong expectations can be used to predict this amount. In effect, the normalization in this method is to a time period (24 h). A directly analogous means of normalizing a urinary mRNA target— to 24 h or a shorter timeframe— seems reasonable if the RNA analyte of interest is sufficiently stable to be reliably quantified after many hours of the refrigeration typically used in 24-h urine collections.

#### 2.1.2 Using urinary creatinine to normalize for changes in urine osmolality

A breakthrough in the normalization of urinary proteins came from a simple insight. Albumin is excreted at a steady rate, as is creatinine. Therefore, Ginsberg et al. predicted that the ratio of albumin to creatinine in any urine sample collected from a single voiding episode (a so-called “spot urine sample”) would be proportional to the ratio found in a complete 24-h collection. [11] Their data and much subsequent data have supported this intuition. The use of an albumin-to-creatinine ratio in a spot urine is appealing for two reasons: it is simpler compared to collecting urine for 24 h, and it is likely more accurate because it does not rely on a calculated estimate of predicted 24-h urinary creatinine excretion and is independent of the accuracy with which collection durations are reported. We have published results of pre-amplified qRT-PCR for gene targets in human urine supernatant and their relationship with urinary creatinine. In that cross-over study of renal physiology during low-salt diet and after saline infusion, we did not find a major or consistent relationship between urinary creatinine and unadjusted Ct values [8]. The participants were relatively young and healthy, and they served as their own controls. Whether the lack of explanatory value of the urinary creatinine will generalize to other studies remains to be seen.

#### 2.1.3 Using urine specific gravity to normalize for changes in urine osmolality

Beyond urinary creatinine, urinary specific gravity has been used to account for differences in urine osmolality in the context of some urinary biomarkers [16,17]. Despite being simple to measure, specific gravity has not been used to normalize urinary extracellular mRNA biomarkers to our knowledge. This approach has the limitation of not having been widely adopted for normalization of proteins or other traditional biomarkers. Not surprisingly, urinary osmolality itself has also been proposed as a normalizer for urinary biomarkers in some contexts such as metabolomics. This approach has not been applied to urinary extracellular mRNA to our knowledge [18].

### 2.2 Normalization for widespread changes in the secretion of extracellular mRNA into the urine

It would be sensible to seek alternatives to urinary creatinine for normalizing urinary biomarkers. It is inherently more complex to measure a second analyte type and an RNA molecule in order to assay the RNA molecule than to compare the relative expression of two or more RNA molecules. The following are several approaches to normalization using an RNA molecule that have a rationale. One could normalize to genes expressed in cells directly relevant to the pathophysiology of interest but unlikely to be affected by that pathophysiology. The magnitude of the target biomarker signal thus would be placed in the seemingly reasonable context of the proportional contribution of the specific cell type to the total mixture of urinary extracellular mRNA molecules. Our impression is that this reasoning underlies one group’s reported success [7]. One could also explore normalization using genes ubiquitously expressed in cells and expressed to a similar degree across cell types, an approach used in qRT-PCR of solid tissues. If abundance in the extracellular compartment reflects abundance of the transcripts inside cells adjacent to that compartment, this strategy might be expected to be effective. In addition, these purely RNA-based strategies might successfully address the concern about incomparability between individuals due to differences in urine flow rate, the rate at which blood is processed into urine— a rate affected by how much water has been consumed recently. The urine flow rate might be expected to equally affect the urinary concentration of many transcripts, including biomarker transcripts and transcripts used for normalization. However, this approach has not been tested extensively and these assumptions have thus not yet been sufficiently explored. In addition, one could normalize to the number of extracellular vesicles per volume of urine as assessed by protein markers, flow cytometry [19], nanoparticle tracking analysis [20,21], or other means of estimating. The most surprising possibility is that normalization to assay urine input volume might be sufficient. Seo et al. extracted total mRNA from 50 mL of urine, and in that sense their study differs from studies of urinary extracellular mRNA. In any event, they concluded that both absolute and relative quantification (relative to 18S rRNA) of biomarkers are useful for non-invasive diagnosis of acute kidney allograft rejection [22]. However, this approach does not account for the variability in urine flow rate between individuals and within an individual under different patterns of drinking fluids or differences in kidney function. As we have previously mentioned, under various pathophysiological conditions the rate of urine production is altered and thus the concentration of vesicles in two equal aliquots of urine might be different due to different rates of production of urine. Additionally, Ben-Dov et al. have reported that when total RNA was extracted from extracellular vesicles (of uncertain purity) isolated from equal volumes of urine, they observed variability in RNA yield between individuals as well as between genders [23].

## 3 Conclusion

Measuring urinary extracellular mRNA to understand human health builds on long tradition. The history of urinary diagnostic testing spans from diagnosis of diabetes mellitus by tasting urine for unusual sweetness to the use of urinary minerals (e.g., sodium) or proteins (e.g., albumin) to characterize disease to the current exploration of urinary extracellular mRNA. To advance the state of the art further will require dedicated experiments based on first principles to identify a generalizable strategy for normalizing urinary extracellular mRNA biomarkers. Ironically, pending new investigations, the most universally reasonable molecule based on first principles is likely urinary creatinine despite our intuition that this might not be the *optimal* choice for any particular application. A bespoke selection of an mRNA molecule expressed by the cells of interest and not dysregulated by the pathology of interest has the theoretical advantages over creatinine of being able to account for a global increase in the export of vesicles as well as making their measurement more practical by limiting analysis to one type of analyte, mRNA.

## Conflict of interest

JBB is an inventor of a provisional patent related to detection of mineralocorticoid receptor activation using urinary extracellular mRNA.

## Funding sources

JBB is funded by the National Institutes of Health, grant number K23-HL128909. Dr. Byrd is co-investigator on research funded by Apple to understand trajectories of health. JML is funded by the National Institutes of Health, grant number 1R01DK117875.
